# Factors associated with clinically significant nephritis in systemic lupus erythematosus patients who present with glomerular hematuria: a cross-sectional study

**DOI:** 10.1007/s00296-026-06264-4

**Published:** 2026-08-07

**Authors:** Konstantinos Drougkas, Eda Otman, Spyridon Katechis, Maria Pappa, Ozge Yogurtcu, Sofia Flouda, Petros Nikolopoulos, Sophia Lionaki, Aggelos Banos, George Liapis, George Bertsias, Dimitrios T. Boumpas, Antonis Fanouriakis

**Affiliations:** 1 Rheumatology and Clinical Immunology Unit, Medical School, Attikon University Hospital, National and Kapodistrian University of Athens, 1 Rimini Stree, Athens, 12462 Greece; 2 Department of Internal Medicine, Division of Rheumatology, Manisa State Hospital, Manisa, Turkey; 3 Department of Internal Medicine, Division of Rheumatology, Izmir Katip Celebi University, Izmir, Turkey; 4 Department of Nephrology, 2nd Propaedeutic Internal Medicine, Medical School, Attikon University Hospital, National and Kapodistrian University of Athens, Athens, Greece; 5 First Department of Pathology, Laiko General Hospital, School of Medicine, National and Kapodistrian University of Athens, Athens, Greece; 6 Rheumatology, Clinical Immunology and Allergy Department, University Hospital of Heraklion, University of Crete, Heraklion, Greece

**Keywords:** Systemic lupus erythematosus, Lupus nephritis, Kidney biopsy, Glomerular hematuria

## Abstract

EULAR recommends kidney biopsy in systemic lupus erythematosus (SLE) patients with glomerular hematuria, whereas both ACR and KDIGO require the presence of proteinuria and/or impaired kidney function. We explored the value of hematuria for the diagnosis of new-onset lupus nephritis (LN). Cross-sectional study of SLE patients who underwent diagnostic kidney biopsy in two independent centers. Clinically significant LN was defined as ISN/RPS class III, IV, V, or mixed III/IV + V. Presentation patterns were categorized by glomerular hematuria, proteinuria, and impaired kidney function. Among patients with glomerular hematuria, regression models identified factors associated with clinically significant LN, and a nomogram was constructed. Of 227 biopsies, 181 (79.7%) showed clinically significant LN. Isolated glomerular hematuria was evident in 11 patients, of whom 9 revealed non-LN pathology; the two LN cases (class II and III) both demonstrated concurrent SLE serologic activity. Among patients with glomerular hematuria (*n* = 137), independent factors associated with clinically significant LN included anti-dsDNA positivity (OR 12.00, 95% CI 3.36–51.40), higher non-renal SLEDAI score (OR 1.20 per point, 95% CI 1.02–1.46), higher proteinuria (OR 3.77 per 1 g/d, 95% CI 2.00–9.03), and younger age (OR 0.96 per year, 95% CI 0.91–1.00). The combined presence of these factors yielded a predicted probability of 97% (95% CI 90–99%) of clinically significant LN, whereas their absence was associated with a probability of only 6% (95% CI 1–23%). When considering the need for kidney biopsy in patients with lupus, hematuria should be evaluated in the context of overall disease activity; extra-renal and serologic activity substantially increase the likelihood of clinically significant LN.

**Supplementary Information:**

The online version contains supplementary material available at 10.1007/s00296-026-06264-4.

## Introduction

Systemic lupus erythematosus (SLE) is a complex multisystem autoimmune disease, characterized by a wide range of manifestations, ranging from mild mucocutaneous and musculoskeletal symptoms to life-threatening organ involvement [1]. Lupus nephritis (LN) is one of the most frequent and severe manifestations of SLE, affecting approximately 30–60% of patients [2]. Despite the development of comprehensive management guidelines and validated treatment targets [3–5], LN patients still experience increased morbidity and mortality [6]. Therefore, prompt identification of SLE patients with signs of kidney involvement is crucial for optimal long-term prognosis.

Recent studies suggest that clinical presentation of LN has become less severe over the years, with a notable decrease in severe and/or rapidly progressive forms, as well as higher estimated glomerular filtration rate (eGFR) levels and more frequent isolated urinary abnormalities at diagnosis, most possibly due to increased physician awareness [7]. However, the correlation between clinical manifestations and kidney histology in SLE is not always accurate, thus a kidney biopsy is still considered indispensable for diagnosis, because histopathologic features guide treatment decisions [8]. Accordingly, rheumatology and nephrology societies, including the European Alliance of Associations for Rheumatology (EULAR), the American College of Rheumatology (ACR) and Kidney Disease: Improving Global Outcomes (KDIGO), have recommended indications to perform a kidney biopsy in patients with SLE [3–5]. While these indications largely reflect the well-established clinical context that guides the decision to perform a kidney biopsy, a couple of notable differences exist. For instance, EULAR recommends a kidney biopsy in SLE patients with any sign of kidney involvement, including isolated glomerular hematuria [3], whereas both ACR and KDIGO refer to the presence of proteinuria and/or impaired kidney function to justify a kidney biopsy [4,5]. Furthermore, in patients with SLE and clinical evidence of kidney involvement, additional diagnoses other than LN may exist, including but not limited to focal segmental glomerulosclerosis (FSGS) and IgA nephropathy [9]. Finally, kidney biopsy is an invasive procedure that, albeit generally safe, is not devoid of risks and may also not be available in all clinical settings [10].

Based on the above, we sought to explore the various renal presentation patterns and respective kidney biopsy results in patients with SLE, with a special focus on glomerular hematuria, in a cross-sectional study performed in two independent centers. Based on our results, we developed a multivariable model to estimate probability of clinically significant LN among SLE patients who develop glomerular hematuria.

## Methods

### Study population

In this cross-sectional study, data of SLE patients from two academic centers (Rheumatology and Clinical Immunology Unit, Attikon University Hospital, Greece, and Division of Rheumatology, Izmir Katip Celebi University, Turkey), who underwent a first kidney biopsy due to suspicion for LN between 2000 and 2025, were collected. SLE diagnosis was established according to the existing disease classification criteria in each time period [11–13]. Patients were excluded if they had (i) kidney biopsy reports with missing data or biopsies containing less than 10 glomeruli, or (ii) key clinical variables missing from their medical records.

The study was approved by the Ethics Committee of Attikon University Hospital (protocol number 103/06-03-2014). As the study relied on routinely collected and de-identified data, informed consent from patients was not required.

### Data collection and definitions

Clinical and laboratory data at the time of kidney biopsy were extracted from hospital medical records, including demographics (age, sex and disease duration), SLE disease activity assessed by the Systemic Lupus Erythematosus Disease Activity Index 2000 (SLEDAI-2 K) [14], presence of new-onset hypertension, serum creatinine and eGFR [based on Chronic Kidney Disease Epidemiology Collaboration (CKD-EPI formula)], 24-hour urinary protein, glomerular hematuria, urinary casts, complement levels and anti- double stranded DNA (anti-dsDNA) antibodies [treated as binary variables - low C3 and/or low C4 vs. both normal, anti-dsDNA (ELISA or CLIFT assay)].

Renal presentation patterns at the time of biopsy were categorized based on the presence of glomerular hematuria, proteinuria, impaired kidney function, and combinations thereof. Categories were mutually exclusive and defined according to all possible combinations of these three variables present at the time of biopsy. Glomerular hematuria was defined as > 5 red blood cells (RBC)/high power field, with ≥ 40% dysmorphic RBCs or ≥ 5% acanthocytes. Proteinuria was defined as proteinuria ≥ 0.15 g/24 h or urine protein-creatinine ratio (UPCR) ≥ 150 mg/g, while impaired kidney function was defined as eGFR < 60 mL/min/1.73 m². All manifestations were new-onset, with no evidence of pre-existing abnormalities. When present, all the above findings were confirmed with repeat testing prior to kidney biopsy. Urine sediment examination was performed as part of routine clinical care in both centres. Medical records were reviewed to exclude non-glomerular causes of hematuria, including radiological evidence of urolithiasis, positive urine cultures consistent with urinary tract infection, active menstruation at the time of urine sampling, and documented urological malignancy.

Kidney biopsy data were collected from pathology reports, which were evaluated by experienced nephropathologists in both centres, according to the International Society of Nephrology/Renal Pathology Society (ISN/RPS) 2003 classification system [15]; in patients with diagnoses other than LN, the specific histological diagnosis was recorded. Clinically significant LN was defined as LN class III, IV, V or mixed III/IV + V, encompassing the main LN classes that typically necessitate immunosuppressive treatment; class I-II LN and non-LN kidney pathologies comprised the comparator group.

### Statistical analysis

Demographic, clinical, laboratory data, and kidney biopsy results are summarized using descriptive statistics. Continuous variables are expressed as means with standard deviations or medians with interquartile ranges, depending on data distribution. Categorical variables are expressed as frequencies and percentages.

Potential factors associated with clinically significant LN were evaluated in the subgroup of SLE patients with glomerular hematuria. Univariable logistic regression analysis was performed and variables with a p-value < 0.2, as well as those considered clinically relevant, were candidates for multivariable modeling. A forward stepwise selection procedure was employed, with variable retention based on the likelihood ratio test (LRT) and the Akaike information criterion (AIC). To avoid multicollinearity among associated variables, the non-renal clinical SLEDAI (cSLEDAI) - excluding renal and serologic components - was used instead of the total SLEDAI-2 K score in the analyses. For clinical application, a nomogram was constructed to illustrate the final model. The discriminative ability of the final model was assessed using the area under the receiver operating characteristic curve (AUC), with a 95% confidence interval derived from 2,000 bootstrap resamples. Hosmer-Lemeshow goodness-of-fit test was used for the evaluation of model fit. Calibration performance, which describes how closely the calculated probabilities agree with the observed outcomes, was examined by Brier score, calibration-in-the-large, calibration slope. Calibration curves were constructed to visually assess model performance. Internal validation was performed using bootstrap resampling (1000 resamples) to estimate bias-corrected calibration performance. Sensitivity analyses were performed, additionally adjusting for treating center and calendar period of kidney biopsy (≤ 2016 vs. >2016), after excluding patients with pure Class V lupus nephritis, and using Firth’s penalized (bias-reduced) logistic regression to assess robustness to potential small-sample bias in coefficient estimation. Based on the negligible proportion of missing data (< 5%) for crucial variables, no imputation techniques were employed.

All analyses were performed using R Statistical Software (v4.5.0; R Foundation for Statistical Computing, Vienna, Austria).

## Results

### Cohort characteristics

In total, 227 SLE patients who underwent a diagnostic kidney biopsy were included. Median (IQR) age at the time of biopsy was 36 (23) years; 44 patients (19.4%) underwent biopsy after 50 years of age. Detailed patient characteristics are presented in Table 1. Clinical signs of kidney involvement were present at SLE diagnosis in half of the patients (50.7%), while 55 (24.2%) developed such signs more than 5 years following disease diagnosis. Median (IQR) SLEDAI-2 K score at time of biopsy was 14 (8) and hypertension was present in 22.5% of patients. Glomerular hematuria was observed in 137 (60.4%) patients, while median (IQR) proteinuria was 1.9 (2.5) g/day, with 71 patients (31.3%) exhibiting nephrotic range proteinuria. Median (IQR) eGFR was 98 (62) mL/min/1.73 m² and 23 patients (10.1%) presented with eGFR < 30 mL/min/1.73 m².

**Table 1:** Demographic, clinical and histological characteristics of patients (*n* = 227)

Characteristics	Median (IQR) or *n* (%)
**At kidney biopsy**	
Female Sex, n (%)	186 (81.9)
Αge, median (IQR)	36.39 (23)
SLE duration prior to kidney involvement, years, median (IQR)	0.08 (4.8)
Kidney involvement at SLE diagnosis, n (%)	115 (50.7)
Anti-dsDNA, n (%)	140 (61.7)
Low C3/C4, n (%)	168 (74)
SLEDAI-2 K, median (IQR)	14 (8)
Hypertension, n (%)	51 (22.5)
Glomerular hematuria, n (%)	137 (60.4)
Urinary casts, n (%)	24 (10.6)
Serum creatinine, median (IQR)	0.8 (0.7)
eGFR, median (IQR)	98 (62)
Proteinuria (g/d), median (IQR)	1.89 (2.5)
**Kidney biopsy result**	
Class II	10 (4.4)
Class III	62 (27.3)
Class IV	44 (19.4)
Class V	46 (20.3)
Class III/IV + V	29 (12.8)
No LN	36 (15.9)

Continuous variables are presented as median (interquartile range, IQR) and categorical variables as absolute number (percentage), as appropriate. Clinical characteristics at LN diagnosis and renal biopsy results are shown. Data were collected retrospectively from medical records. LN: lupus nephritis, anti-dsDNA: anti- double stranded DNA, SLEDAI-2 K: systemic lupus erythematosus disease activity index 2000, eGFR: estimated glomerular filtration rate

Regarding histopathology, 181/227 biopsies (79.7%) revealed clinically significant LN: class III 62 (27.3%), class IV 44 (19.4%), class V 46 (20.3%), and class III/IV + V 29 (12.8%). Moreover, 10 patients (4.4%) had class II LN, and 36 patients (15.9%) were found to have pathology other than LN. The majority of non-LN cases revealed FSGS (*n* = 13, 36.1%), followed by IgA nephropathy (*n* = 4, 11.1%) and non-specific lesions (*n* = 4, 11.1%); a complete list of non-LN diagnoses is shown in **Supplementary Table **S1.

### Glomerular hematuria in the absence of other signs of lupus activity may lower the yield of clinically significant LN

**Fig. 1:**
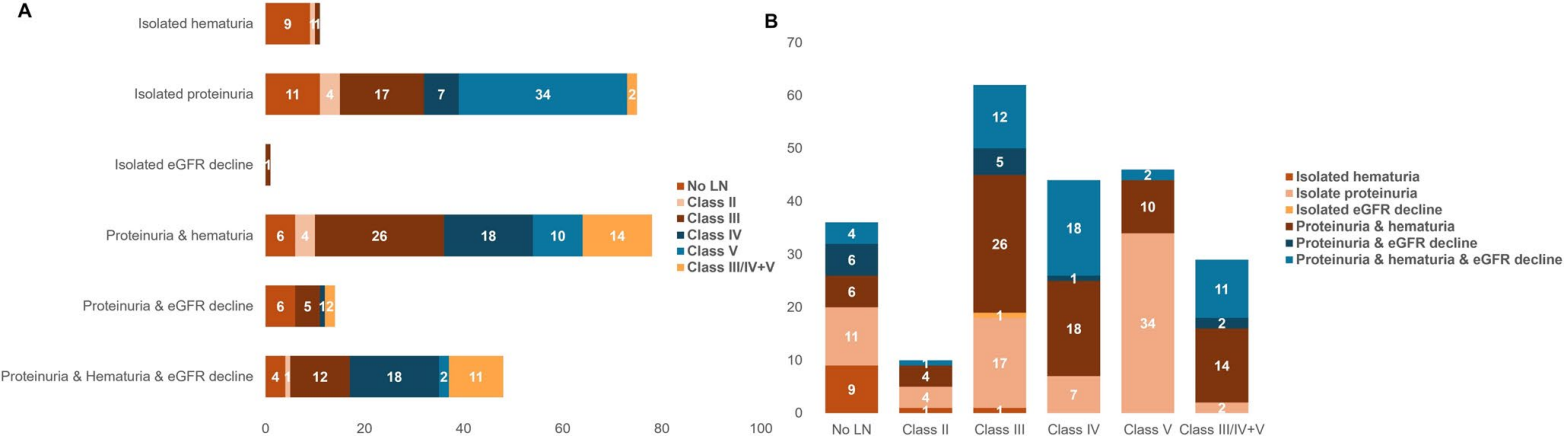
Relationship between renal presentation patterns and kidney biopsy results in SLE patients. **A** Distribution of kidney biopsy results across different clinical presentation patterns at the time of biopsy. Each horizontal bar represents a renal presentation pattern (e.g., isolated hematuria, isolated proteinuria, combined abnormalities), and the total length of the bar corresponds to the number of patients in that category. Colored segments within each bar indicate the number of patients with different biopsy results (LN class II–V or non-LN pathology). **B** Distribution of renal presentation patterns across each kidney biopsy result. Each vertical bar represents a biopsy result (LN class II–V or non-LN pathology), and colored segments show the proportion of patients presenting with each renal presentation pattern. This bidirectional display illustrates both how clinical presentation relates to histological findings and how each biopsy result typically manifests clinically. SLE: systemic lupus erythematosus, LN: lupus nephritis

Renal presentation patterns at the time of kidney biopsy are shown in Fig. 1. The most frequent presentation was combined proteinuria and hematuria (*n* = 78), followed by isolated proteinuria (*n* = 75) and a combination of proteinuria, hematuria, and impaired kidney function (*n* = 48). Less common presentations included isolated hematuria (*n* = 11), proteinuria and impaired kidney function (*n* = 14), and isolated impaired kidney function (*n* = 1).

Descriptive analysis of biopsy results across the various clinical presentation patterns revealed several trends (Fig. 1A). As expected, patients who manifested isolated proteinuria (i.e., in the absence of glomerular hematuria and impaired kidney function) mostly revealed class V LN (34/75, 45.3%), whereas among the 126 patients who presented with combined hematuria and proteinuria (with or without impaired kidney function), 99 (78.6%) had proliferative or mixed LN (Class III 38/126, 30.2%; Class IV 36/126, 28.6%; Class III/IV + V 25/126, 19.8%). Vice versa, looking at kidney biopsy results (Fig. 1B), class V LN predominantly presented with isolated proteinuria (34/46, 73.9% of class V LN), while among the 135 patients with proliferative LN, 99 (73.3%) presented with combined abnormalities: 38/62 (61.3%) of class III, 36/44 (81.8%) of class IV, and 25/29 (86.2%) of class III/IV + V.

Notably, patients presenting with isolated glomerular hematuria, albeit representing only a small group (*n* = 11), often revealed kidney pathology other than LN (9/11), mainly IgA nephropathy (3/9); only one patient had clinically significant LN (class III), while the final patient had class II LN. Importantly, both cases whose biopsies yielded LN had evidence of lupus serologic activity at the time of biopsy, with anti-dsDNA antibody positivity and low complement levels. Conversely, hypocomplementemia and anti-dsDNA positivity were absent in almost all patients with isolated glomerular hematuria, whose biopsy findings indicated kidney pathology other than LN (8/9). Notably, none of these patients presenting with isolated glomerular hematuria developed additional renal abnormalities, including new-onset proteinuria or impaired kidney function, later on during a median (IQR) 3.8 (1.3–4.6) years of follow-up. No patient has been lost to follow-up.

### Factors associated with clinically significant LN among patients with glomerular hematuria

Based on the finding that isolated glomerular hematuria was infrequently associated with LN, we sought to identify the clinical and serological features that increase the probability of clinically significant LN among patients with SLE who present with glomerular hematuria. Of the 137 SLE patients with glomerular hematuria, 112 (81.8%) had clinically significant LN, and 25 (18.2%) did not. In univariable analysis, younger age, anti-dsDNA antibody positivity, low complement levels, higher non-renal cSLEDAI scores, and higher-grade proteinuria were all associated with increased odds of clinically significant LN (Table 2). In multivariable analysis, younger age, anti-dsDNA positivity, higher non-renal cSLEDAI scores, and proteinuria remained independently associated with the outcome. Specifically, patients with hematuria and anti-dsDNA antibodies had 12-fold higher odds of clinically significant LN (OR 12.00, 95%CI 3.36–51.4), compared to those without anti-dsDNA. Higher-grade proteinuria and increased extra-renal disease activity were also independently associated with increased likelihood; respective OR (95% CI) were 3.77 (2.00-9.03) for each 1 g/day increase in proteinuria, and 1.20 (1.02–1.46) for each 1-point increase in non-renal cSLEDAI. Older age was associated with decreased likelihood of clinically significant LN, with each additional year conferring a 4% decrease in the odds (OR 0.96, 95% CI 0.91-1.00, Table 2).

**Table 2:** Baseline factors associated with clinically significant LN among patients who present with glomerular hematuria

Variable	Univariable OR (95% CI), *P*-value	Multivariable OR (95% CI), *P*-value
Age (per 1-year increase)	**0.95 (0.92–0.98)**,** < 0.001**	**0.96 (0.91-1.00)**,** 0.05**
Male Sex	0.82 (0.29–2.69), 0.72	
SLE duration prior to kidney involvement	0.94 (0.87–1.02), 0.12	
Kidney involvement at SLE diagnosis	2.13 (0.88–5.41), 0.10	
Anti-dsDNA	**12.00 (4.40–38.8)**,** < 0.001**	**12.00 (3.36–51.45)**,** < 0.001**
Low C3/C4	**4.25 (1.68–10.8)**,** 0.002**	
Non-renal cSLEDAI (per 1 point increase)	**1.20 (1.05–1.39)**,** 0.01**	**1.20 (1.02–1.46)**,** 0.05**
Hypertension	1.21 (0.44–3.91), 0.73	
Serum creatinine (per 1 mg/dL increase)	1.83 (1.05–4.15), 0.08	
eGFR (per 1 mL/min/1.73 m² increase)	1.00 (0.99–1.01), 0.48	
Proteinuria (per 1 g/d increase)	**3.86 (2.09–8.58)**,** < 0.001**	**3.77 (2.00-9.03)**,** < 0.001**
Urinary casts	1.69 (0.52–7.61), 0.43	

Statistical significance (*P* < 0.05) is shown in bold

*SLE* systemic lupus erythematosus, *LN* lupus nephritis, *anti-dsDNA* anti- double stranded DNA, *cSLEDAI*: clinical systemic lupus erythematosus disease activity index, *eGFR*: estimated glomerular filtration rate

The final multivariable model demonstrated excellent discriminative ability, with an AUC of 0.94 (95% CI: 0.89–0.97) (**Supplementary Figure **S1). Hosmer-Lemeshow goodness-of-fit testing indicated adequate model fit (χ² = 3.01, df = 8, *p* = 0.93). Calibration metrics showed a good agreement between predicted and observed outcomes: Brier score was 0.071, calibration-in-the-large was 0.000, and calibration slope was 1.000. Calibration curves of the multivariable model both in the original dataset and in 1000 bootstrap resamples are shown in **Supplementary Figure **S2. Effect estimates for all factors remained directionally consistent in sensitivity analyses (i) adjusting for center and calendar period, (ii) excluding patients with pure Class V lupus nephritis, and (iii) using Firth’s penalized logistic regression (**Supplementary Figure ****S3**).

**Fig. 2:**
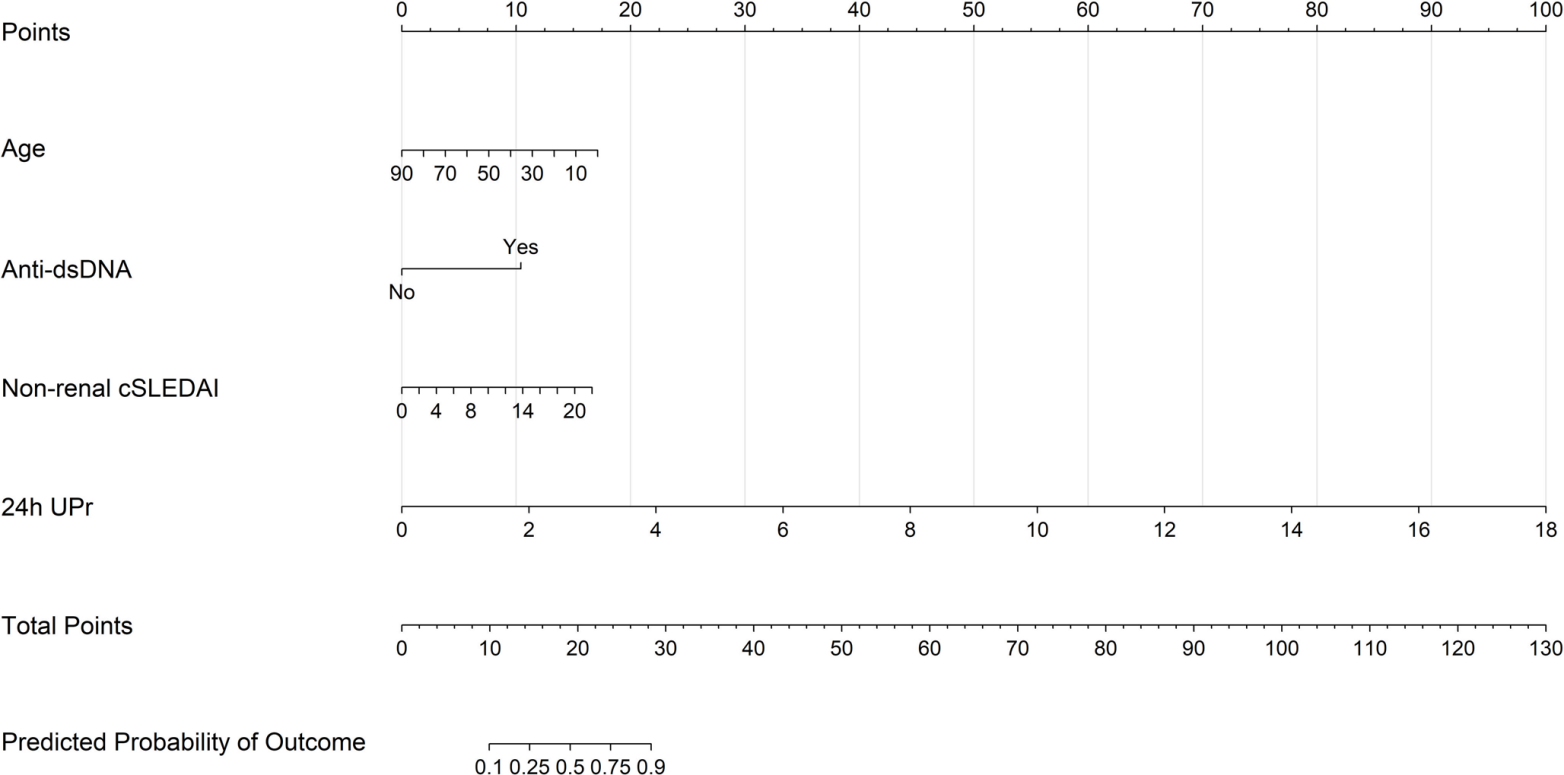
Nomogram for estimating the probability of clinically significant LN among SLE patients presenting with glomerular hematuria. The nomogram provides an individualized probability of clinically significant LN based on age, anti-dsDNA antibody status, proteinuria (g/d), and extra-renal activity. For each variable a corresponding number of points is assigned by projecting vertically to the “Points” axis at the top of the figure. The points for all variables are summed to generate a total score, which is then projected downward to estimate the predicted probability of clinically significant LN. Higher total points indicate higher predicted probabilities. SLE: systemic lupus erythematosus, LN: lupus nephritis, cSLEDAI: clinical systemic lupus erythematosus disease activity index, 24 h UPr: urinary protein (g/d)

**Fig. 3:**
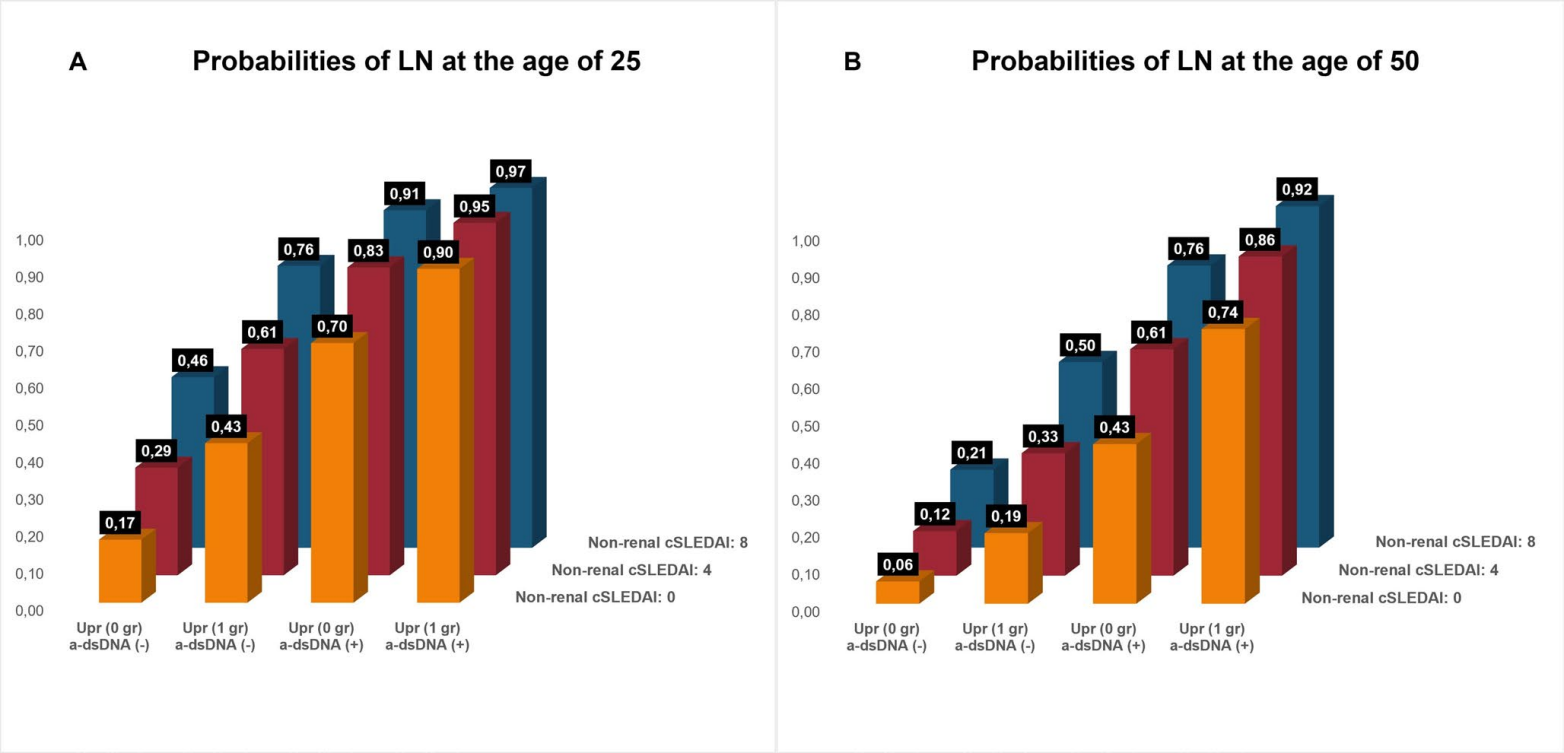
Additive effect of clinical and serological variables on the probability of clinically significant LN among SLE patients who present with glomerular hematuria. Predicted probabilities of clinically significant LN derived from the nomogram model are shown for different combinations of non-renal cSLEDAI scores (0, 4, and 8), proteinuria (0–1 g/d), and anti-dsDNA positivity (+/–), for patients aged 25 **A** and 50 **B** years. Increasing non-renal cSLEDAI scores, proteinuria, and anti-dsDNA positivityare associated with progressively higher predicted probabilities of clinically significant LN, particularly in younger patients. The reference clinical scenario defined by absence of proteinuria, anti-dsDNA negativity, and non-renal cSLEDAI score of 0, corresponds to the lowest predicted probability. SLE: systemic lupus erythematosus, LN: lupus nephritis, cSLEDAI: clinical systemic lupus erythematosus disease activity index

A nomogram was constructed based on the four independent factors from the multivariable model to provide an individualized probability of clinically significant LN among SLE patients who develop glomerular hematuria (Fig. 2). Among patients aged 25 years, the combination of positive anti-dsDNA antibodies, proteinuria of 1 g/d and a non-renal cSLEDAI score of 8, yielded a predicted probability of 97% (95% CI: 90–99%) for clinically significant LN. On the contrary, in patients with isolated glomerular hematuria that lacked all these associated factors at the time of kidney biopsy, the model-estimated probability of clinically significant LN was low (6%, 95% CI: 1–23%) (Fig. 3). A similar trend, although at lower cumulative probabilities, was seen in patients at 50 years of age. Collectively, among patients with SLE who present with glomerular hematuria, the combination of younger age, higher extra-renal disease activity, anti-dsDNA positivity, and higher levels of proteinuria is associated with an increased likelihood of clinically significant LN.

## Discussion

In this cross-sectional study, we associated kidney histology with clinical presentation patterns in patients with SLE, aiming to explore the independent diagnostic value of glomerular hematuria and identify baseline factors associated with clinically significant LN among SLE patients who present with hematuria.

Notably, in our cohort of 227 SLE patients who underwent a first kidney biopsy, approximately 16% revealed a pathology other than LN, with FSGS being the most prevalent alternative diagnosis. Previous studies have reported non-LN diagnoses in 5–16% of kidney biopsies, similarly identifying FSGS as the most prevalent non-LN diagnosis [16,17]. Beyond FSGS, a wide spectrum of other pathologies has been described, including but not limited to thrombotic microangiopathy (TMA), IgA nephropathy, amyloidosis, thin basement membrane and minimal change disease [18,19]. These findings, consistent with our data, highlight the fact that kidney disease in SLE does not equal LN, as distinct or coincident pathologies may exist. These conditions can either be associated with SLE (such as TMA, FSGS and tubulointerstitial nephritis), represent other autoimmune glomerulopathies [IgA nephropathy, anti-glomerular basement membrane (anti-GBM) disease and pauci-immune glomerulonephritis], or be due to non-autoimmune diseases, such as metabolic, hypertensive, or hereditary nephropathies [9]. Distinguishing LN from these diseases is of great importance, in order to prevent the use of inappropriate immunosuppressive treatment. This underlines once more the paramount role of kidney biopsy in SLE, as well as the value of clinical information; absence of systemic and serologic disease activity, presence of atypical autoantibodies, or family history of kidney disease may point towards an alternative diagnosis.

Over the past decades, the clinical presentation of LN at the time of kidney biopsy has evolved towards milder phenotypes. An Italian multicenter cohort spanning the period 1970 to 2016 reported a significant increase in patients presenting with isolated urinary abnormalities, alongside a decrease in nephritic syndrome and rapidly progressive glomerulonephritis [20]. Similarly, a large Spanish study showed that kidney function at the time of biopsy has gradually improved, proteinuria levels have decreased, and LN is increasingly diagnosed in older patients [21]. Together, these studies suggest that earlier recognition of SLE and increased awareness of kidney involvement have contributed to identifying the latter at earlier and less severe stages. Although our study was not designed to explore such temporal trends, patients at kidney biopsy had largely preserved kidney function, with only 10% presenting with eGFR < 30 mL/min/1.73 m². Additionally, approximately 30% of patients exhibited nephrotic range proteinuria, while combination of hematuria and proteinuria (with preserved eGFR) was the most frequently observed clinical pattern. These findings corroborate the notion that at present LN is often detected at earlier stages, before the development of severe nephrotic/nephritic syndrome or rapidly progressive glomerulonephritis.

Urine sediment examination is traditionally used in clinical practice for screening and monitoring disease activity in LN. Yet, the significance of isolated glomerular hematuria – in the absence of proteinuria or eGFR compromise – as a manifestation of LN and whether it per se justifies performing a kidney biopsy remains a subject of debate. Older data from the Toronto Lupus Cohort suggested that isolated hematuria could be an indicator of underlying LN [22]. Among 926 patients with SLE followed between 1970 and 2000, one-third experienced at least one episode of isolated hematuria, while kidney biopsies performed in 22 patients revealed that nearly all (96%) had LN, with 41% showing clinically significant classes. These findings implied that, even in the absence of other abnormalities, hematuria might reflect underlying nephritis. However, the majority of patients with hematuria in this study did not actually undergo kidney biopsy, while 77% of them also had concurrent non-renal manifestations, suggesting generalized lupus activity. A follow-up study from the same center focused on isolated hematuria without other active SLE features; although most patients later manifested additional urinary abnormalities, only 3 of 32 finally underwent biopsy, not allowing solid conclusions about underlying kidney pathology [23]. Our cohort included biopsy data for 11 patients presenting with isolated glomerular hematuria. LN was present in only 2 cases (one class II and one class III), both of whom had additional serologic evidence of lupus activity, with anti-dsDNA antibody positivity and low complement levels. Furthermore, in our analysis, hematuria alone lacked diagnostic value, unless accompanied by other signs of renal or extra-renal disease activity. Specifically, younger patients with anti-dsDNA antibody positivity, higher proteinuria levels, and extra-renal disease activity were significantly more likely to have clinically significant LN (predicted probability of 97%).

The 2025 EULAR recommendations for the management of SLE with kidney involvement include glomerular hematuria in the indications to perform a kidney biopsy, stating that SLE patients presenting with “any sign of kidney involvement” are candidates for kidney biopsy, including glomerular hematuria [3]. They nevertheless specifically add that “the decision to perform a kidney biopsy should consider all clinical factors (including extrarenal disease activity) and laboratory data (such as lupus serology) in each patient”. Our findings align with this holistic approach: hematuria should be interpreted in the context of other clinical and serologic features of disease activity. Specifically, we showed that, when glomerular hematuria is accompanied by extra-renal or serologic activity, the probability of clinically significant LN is substantial, reinforcing the value of kidney biopsy even when hematuria represents the sole renal manifestation. On the other hand, in patients presenting with isolated glomerular hematuria without any other signs of lupus activity (renal, extra-renal, or serologic), particularly in older individuals, the predicted probability of clinically significant LN is lower, though not negligible (~ 6%). In such instances, the decision between performing a kidney biopsy versus vigilant monitoring should be made on a case-by-case basis.

Our study has several limitations that need to be acknowledged. First, the small number of patients presenting with isolated glomerular hematuria (*n* = 11) limits the generalizability of our findings and highlights the need for larger cohorts to confirm our observations. Also, some patients (*n* = 10) with hematuria– either isolated or with other urinary abnormalities – in our centers did not undergo kidney biopsy due to personal reasons or contraindications, and were therefore not included in the analysis; this affects our results from being applied to all SLE patients with isolated hematuria (or, even more, to all SLE patients in general). Second, as data on treatments at the time of kidney biopsy were incomplete in several medical records, treatment variables were not included in our study. A possible influence of therapy at the time of kidney biopsy on renal parameters cannot be completely excluded, although we partly addressed this by adjusting for disease duration. Last, glomerular hematuria was assessed as part of routine clinical care in both centres and was not “centrally” assessed. Urine sediment evaluation remains operator-dependent, and interobserver agreement - even among experienced nephrologists - is limited, particularly for RBC casts, with concordance reported to be around 60%.^24^ Most SLE/LN studies have similarly not systematically characterized dysmorphic RBCs or cellular casts with well-defined and standardized methods, which may have contributed to variability across findings [25].

In conclusion, our study demonstrated that isolated glomerular hematuria in SLE patients yielded a kidney pathology other than LN in a significant proportion of our small patient sample. Among SLE patients presenting with hematuria, younger age, anti-dsDNA positivity, higher levels of proteinuria and increased non-renal cSLEDAI scores defined a subgroup with a higher probability of LN needing immunosuppressive treatment. Our results highlight the importance of integrating urinary findings with serologic and clinical indicators of disease activity, in a holistic approach to guide the need for kidney biopsy in patients with lupus.

## Supplementary Information

Below is the link to the electronic supplementary material.

Supplementary Material 1

## Data Availability

The data that support the findings of this study are available from the corresponding author upon reasonable request.

## Declarations

### Congress abstract publication

This work was presented during the 5th European Lupus Meeting in Lisbon, Portugal, March 04–07, 2026 (Drougkas K, Otman E, Katechis S, Pappa M, Yogurtcu O, Flouda S, et al. PT2:04 Glomerular hematuria and its association with kidney pathology in systemic lupus erythematosus: a matter of context. Lupus Science & Medicine. 2026;13:. 10.1136/lupus-2026-el.35).
