# Supplementary material for: Factors associated with clinically significant nephritis in systemic lupus erythematosus patients who present with glomerular hematuria: a cross-sectional study
Source: Rheumatol Int. 2026 Aug 7;46(8):233. doi: 10.1007/s00296-026-06264-4 (PMC13451229; doi:10.1007/s00296-026-06264-4)
Supplement: Supplementary file 1 [file 296_2026_6264_MOESM1_ESM.docx]

**Glomerular Hematuria and its Association with Kidney Pathology in Systemic Lupus Erythematosus: A Matter of Context**

Konstantinos Drougkas^1^, Eda Otman^2^, Spyridon Katechis^1^, Maria Pappa^1^, Ozge Yogurtcu^3^, Sofia Flouda^1^, Petros Nikolopoulos^4^, Sophia Lionaki^4^, Aggelos Banos^1^, George Liapis^5^, George Bertsias^6^, Dimitrios T. Boumpas^1^, Antonis Fanouriakis^1^

^1^ Rheumatology and Clinical Immunology Unit, Attikon University Hospital, National and Kapodistrian University of Athens, Medical School, Athens, Greece.

^2^ Manisa State Hospital, Department of Internal Medicine, Division of Rheumatology, Manisa, Turkey.

^3^ Izmir Katip Celebi University, Department of Internal Medicine, Division of Rheumatology, Izmir, Turkey.

^4^ Department of Nephrology, 2nd Propaedeutic Internal Medicine, Attikon University Hospital, Medical School, National and Kapodistrian University of Athens, Athens, Greece.

^5^ First Department of Pathology, Laiko General Hospital, National and Kapodistrian University of Athens, School of Medicine, Athens, Greece.

^6^ Rheumatology, Clinical Immunology and Allergy Department, University Hospital of Heraklion, University of Crete, Heraklion, Greece.

**Corresponding author**: Antonis Fanouriakis

Rheumatology and Clinical Immunology Unit, Attikon University Hospital, 1 Rimini Street, 12462, Athens, Greece

Telephone: +302105831667 - **Mail**: [afanour@med.uoa.gr](mailto:afanour@med.uoa.gr)

**Supplementary Table S1** Detailed kidney biopsy results in SLE patients with non-LN pathology

| **Diagnosis** | **n (%)** |
| --- | --- |
| Thin basement membrane disease | 2 (5.56) |
| Amyloidosis | 1 (2.78) |
| ANCA | 2 (5.56) |
| C3 glomerulopathy | 1 (2.78) |
| Fibrillary glomerulonephritis | 1 (2.78) |
| Focal segmental glomerulosclerosis | 13 (36.11) |
| IgA nephropathy | 4 (11.11) |
| Minimal change disease | 1 (2.78) |
| Membranoproliferative glomerulonephritis | 1 (2.78) |
| Membranous nephropathy | 1 (2.78) |
| Tubulointerstitial nephritis | 3 (8.33) |
| Non-specific lesions | 4 (11.11) |
| Normal | 2 (5.56) |

Data are presented as absolute number (percentage). Percentages are calculated out of the 36 patients with non-LN pathology. Biopsy results are based on pathology reports evaluated by nephropathologists.

**Supplementary Figure S1**. Receiver operating characteristic curve of the multivariable model.

The ROC curve shows the discriminative performance of the final multivariable model in distinguishing patients with clinically significant LN from those without. The model demonstrated excellent discrimination, with an area under the curve (AUC) of 0.94 (95% CI: 0.89–0.97), estimated from 2,000 stratified bootstrap resamples. The dashed diagonal line represents the line of no discrimination (AUC = 0.50).

**Supplementary Figure S2** Calibration curves of the multivariable model.

Calibration curves comparing model-based predicted probabilities of clinically significant LN (x-axis) with observed frequencies (y-axis). The dotted diagonal line represent perfect calibration, where predicted and observed probabilities are identical. The orange solid line represents the apparent performance of the multivariable model in the original dataset, while the red solid line represents the bias-corrected performance based on 1000 bootstrap resamples. The close alignment between the bias-corrected curve and the ideal diagonal line indicates good calibration. Calibration metrics confirm the good agreement between predicted and observed outcomes: Brier score was 0.071, calibration-in-the-large was 0.000, and calibration slope was 1.000.

**Supplementary Figure S3**. Sensitivity analyses for the association of baseline factors with clinically significant LN among patients presenting with glomerular hematuria.

Forest plot showing odds ratios (ORs) with 95% confidence intervals (CIs) for the four factors of the final multivariable model (age, 24-hour proteinuria, non-renal cSLEDAI, and anti-dsDNA antibody positivity), in the main model and four sensitivity analyses: (1) additional adjustment for treating center, (2) additional adjustment for calendar period of kidney biopsy (dichotomized at ≤2016 vs. >2016), (3) exclusion of patients with pure Class V lupus nephritis (n=12), and (4) Firth's penalized (bias-reduced) logistic regression. Point estimates represent odds ratios; horizontal lines represent 95% CIs. The vertical dashed line indicates the line of no effect (OR = 1).

LN: lupus nephritis, anti-dsDNA: anti- double stranded DNA, cSLEDAI: clinical systemic lupus erythematosus disease activity index
